# Concurrent administration of Docetaxel and Stealth® liposomal doxorubicin with radiotherapy in non-small cell lung cancer : excellent tolerance using subcutaneous amifostine for cytoprotection

**DOI:** 10.1038/sj.bjc.6600486

**Published:** 2002-08-12

**Authors:** M I Koukourakis, K Romanidis, M Froudarakis, G Kyrgias, G V Koukourakis, G Retalis, N Bahlitzanakis

**Affiliations:** Tumour and Angiogenesis Research Group, PO Box 12, Democritus University of Thrace, Alexandroupolis 68100, Greece; Department of Radiotherapy and Oncology, Democritus University of Thrace, Alexandroupolis 68100, Greece; Department of Lung Diseases, Venizelion General Hospital, Heraklion, Crete, Greece; Department of Radiotherapy and Oncology, Hellenic Cancer Insitute, Saint Savvas Hospital, Athens, Greece

**Keywords:** radiotherapy, amifostine, stealth liposomal doxorubicin, docetaxel, non-small cell lung cancer

## Abstract

The substantial augmentation of the radiation sequelae during chemo–radiotherapy with novel drugs masks the real potential of such regimens. In this study we examined whether subcutaneous administration of amifostine can reduce the toxicity of a highly aggressive chemo–radiotherapy scheme with Stealth® liposomal doxorubicin (Caelyx®) and Docetaxel (Taxotere®) in non-small cell lung cancer. Twenty-five patients with stage IIIb non-small cell lung cancer were recruited in a phase I/II dose escalation trial. The starting dose of Taxotere® was 20 mg m^−2^ week and of Caelyx® was 15 mg m^−2^ every two weeks, during conventionally fractionated radiotherapy (total dose of 64 Gy). The dose of Taxotere®/Caelyx® was, thereafter, increased to 20/25 (five patients) and 30/25 mg m^−2^ (15 patients). Amifostine 500 mg was given subcutaneously before each radiotherapy fraction, while an i.v. amifostine dose of 1000 mg preceded the infusion of docetaxel. The ‘in-field’ radiation toxicity was low. Grade 3 esophagitis occurred in 9 out of 25 (36%) patients. Apart from a marked reduction of the lymphocyte counts, the regimen was deprived from any haematological toxicity higher than grade 1. No other systemic toxicity was noted. The CR and CR/PR rates in 15 patients treated at the highest dose level was 40% (6 out of 15) and 87% (13 out of 15) respectively. It is concluded that the subcutaneous administration of amifostine during high dose Taxotere®/Caelyx® chemo–radiotherapy is a simple and effective way to render this aggressive regimen perfectly well tolerated, by reducing the systemic and the ‘in-field’ toxicity to the levels expected from simple conventional radiotherapy. The impressive tolerance and the high CR rate obtained encourages the conduct of a relevant randomized trial to assess an eventual survival benefit in patients with non-small cell lung cancer.

*British Journal of Cancer* (2002) **87**, 385–392. doi:10.1038/sj.bjc.6600486 www.bjcancer.com

© 2002 Cancer Research UK

## 

Non-small cell lung cancer (NSCLC) is a major cause of death from cancer. Even in early stages of the disease 30–50% of patients will relapse locally or to distant organs following surgery (Kayser *et al*, 1987). Radiotherapy is the treatment of choice in locally advanced inoperable cases but the results are frustrating as the cure rate is less than 10% (Koukourakis *et al*, 1996). Accelerated and/or hyperfractionation techniques may increase the local control rate by 10–20% (Saunders *et al*, 1996). Concurrent platinum based chemo–radiotherapy showed improved efficacy in randomized studies (Marino *et al*, 1995; Schaake-Koning *et al*, 1992; Jeremic *et al*, 1996), while in a recent study by Furuse *et al* (1999) the concurrent approach yielded enhanced median survival duration in selected patients with unresectable stage III NSCLC.

Several novel anti-cancer drugs have shown important radiosensitizing properties *in vitro* (Hennequin *et al*, 1996; Tamura *et al*, 1997; Milas *et al*, 1999), which encourages clinical trials of chemo–radiotherapy. In phase I/II trials, both paclitaxel and docetaxel showed a high rate of complete responses in locally advanced NSCLC (Reckzeh *et al*, 1996; Koukourakis *et al*, 1998a). However, severe mucositis and immunological toxicity accompanied these regimens (Koukourakis *et al*, 1999a). A novel antimetabolite, Gemcitabine, has shown remarkable activity against NSCLC and important radiosensitizing properties (Milas *et al*, 1999). Clinical trials, however, suggest that substantial reduction on the gemcitabine dose should be considered in order to have acceptable toxicity (Eisbruch *et al*, 2001; Trodella *et al*, 2002). Combination of low dose of camptothecines with radiotherapy also results in a very high mucosal toxicity (Graham *et al*, 1996). Severe mucositis is also the dose limiting toxicity of the combination of radiotherapy with liposomal doxorubicin (Koukourakis *et al*, 1999b).

The dramatic augmentation of the radiation sequelae during chemo–radiotherapy with all these novel compounds masks the real potential of such regimens as (1) only a reduced dose of the chemotherapeutic agent can be safely administered during radiotherapy, (2) rapid repopulation becomes a more common cause of radiotherapy failure as unnecessary delays of the schedule are enforced due to severe toxicity and, (3) combination of more than one of these drugs with radiotherapy is expected to be problematic. Unless effective and selective cytoprotection of normal tissues increases the tolerance, these novel and potentially highly effective chemo–radiotherapy regimens will not find their place in the clinical practice.

In the present dose escalating study we show that standard radiotherapy can be safely combined with high doses of two drugs, namely Stealth® liposomal doxorubicin (Caelyx®) and Docetaxel (Taxotere®), if subcutaneous amifostine is used as a broad spectrum cytoprotective agent (Koukourakis *et al*, 2000a).

## PATIENTS AND METHODS

### Recruitment criteria

Patients with histologically-confirmed inoperable (stage IIIb; T3,4-N2,3-M0) NSCLC entered this phase I/II study. All patients had disease infiltrating the thoracic wall, trachea, thoracic vessels and pericardium, and/or presence of contralateral mediastinal and/or supraclavicular involved nodes. Patients with white blood cells <2500 μl^−1^ and platelets <120 000 μl^−1^ were excluded. Patients with haemoglobin <10 g dl^−1^ were transfused until haemoglobin levels were raised >12 g dl^−1^. Pregnant women or patients with major heart, lung, liver, renal, psychiatric disease, or with haematological malignancies were excluded. Written informed consent was obtained from all patients. The study was approved by the local Oncology Trial Committee.

### Pretreatment and treatment evaluation

Baseline studies included physical examination, chest X-rays, whole blood count with differential and platelet count, complete biochemical profile, bone scan and computed tomography (CT-scan) of the chest and upper abdomen. Whole blood cell count was performed once a week during the radiotherapy period and for 4 weeks thereafter. Serum urea and creatinine, as well as liver enzymes were analysed every 2 weeks during radiotherapy. Electrocardiograms were performed every 3 weeks. Acute radiation toxicity was registered twice weekly. The World Health Organization (WHO) scale was used to assess chemotherapy and acute radiation toxicity (World Health Organization, 1979).

Response to treatment was assessed with a CT-scan on day 25 (to allow eventual modification of the radiotherapy fields) and 45–60 days following treatment completion. Complete response (CR) was defined as the disappearance of a measurable lesion within 2 months following treatment completion, lasting for at least 2 months after response documentation. Remnant scar on CT-scan measuring less than 5% of the initial tumour volume and showing no signs of progression within 2 months after response documentation was considered as complete response. Similarly, partial and minimal response (PR and MR) was defined as a 50–95% and 25–49% reduction in tumour size, respectively. Smaller reductions in tumour size (0–24%) that lasted at least 2 months after response documentation were considered stable disease (SD). All other cases were considered progressive disease (PgD), regardless of the initial response.

The duration of response was measured from the time the criteria for the objective response were first met. CT-scan done every 2 months for the first 6 months and every 3 months (or earlier if necessary) thereafter. The patients were followed with clinical examination and haematological and biochemical tests once every 2 months. Bone scan or computed tomography other than chest was performed when indicated by specific symptomatology. For late radiation sequelae the RTOG scale was used (Rtog late effects working group, 1995).

### Radiotherapy schedule

Radiotherapy treatment planning was based on chest CT-scans. A 6MV LINAC (Philips) was used to treat all patients. Anteroposterior radiation portals encompassing the primary tumour area and part of the mediastinum were used to deliver a daily dose of 2 Gy, up to 44 Gy. In patients with upper lobe tumours, the lower margine of the mediastinal fields were placed 6 cm below the carina, while the upper margin was extended to include the homolateral supraclavicular area. In patients with lower lobe tumours, the upper limit of the mediatinal field was placed to the sterno–clavicular joint level. One or two oblique fields directed to the bulky tumour area were thereafter used to increase the total tumour dose to 64 Gy (dose per fraction 2 Gy). The planned overall treatment time was 6.5 weeks. No conformal techniques were used.

### Chemo–radiotherapy combination and dose escalation

The chemo–radiotherapy scheme used is diagrammatically reported in Table 1

**Table 1:**
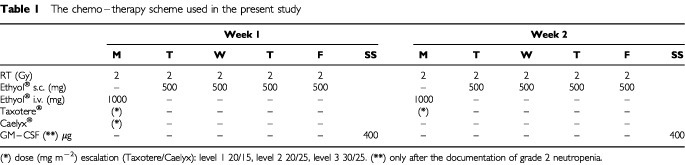
The chemo–therapy scheme used in the present study

. Docetexel (Taxotere®) was given every Monday, once weekly during the seven consecutive weeks of radiotherapy. Stealth liposomal doxorubicine (Caelyx®) was given every other Monday, once every 2 weeks (1st, 3rd, 5th and 7th week of radiotherapy). The starting dose of Taxotere®/Caelyx® regimen was 20/15 mg m^−2^. Five patients were recruited in this dose level. The dose of the drugs was escalated in two higher dose levels : 20/25 and 30/25 mg m^−2^ (five patients in each dose level). The Taxotere® dose of 30 mg m^−2^ week and the Caelyx® dose of 25 mg m^−2^ every 2 weeks were the maximum tolerated doses identified in previously conducted chemo–radiotherapy dose escalation studies (Koukourakis *et al*, 1998a; 1999b). No further escalation beyond this ‘maximum dose level’ was to be tried. Once the MTD (or the maximum dose level) would have been achieved, the study was to continue as a phase II one to assess response and toxicity of the proposed schedule in a cohort of 15 patients.

### Taxotere*®* and Caelyx*®* administration procedure

Twelve hours and 30 min before chemotherapy patients received 32 mg p.o. and 125 mg i.v. bolus methyl-prednisolone, respectively. Ranitidine 300 mg p.o. was given daily throughout the 6 week treatment. Tropisetron (10 mg i.v.) was given as antiemetic treatment. Taxotere® was diluted in 250 ml normal saline and infused within 20 min. Blood pressure and symptomatology assessment was monitored every 5 min during infusion and every 15 min for the following hour. No steroids were used thereafter if no allergic reaction occurred. Whenever allergic reaction was observed patients were given methyl-prednisolone (32 mg p.o.) 12 h after chemotherapy.

Caelyx® was diluted in 250 ml distilled water and infused within 30 min. Blood pressure was monitored and symptoms were assessed every 5 min during infusion and every 15 min for the following hour.

### Administration of amifostine

During the days of chemo–radiotherapy (Mondays), amifostine was given intravenously immediately before docetaxel. Amifostine (Ethyol®), 1000 mg diluted in 50 ml normal saline, was infused within 5 min, the patient being in a supine position, and under continuous monitoring of blood pressure. Docetaxel administration began 10 min after the amifostine infusion.

During the days of radiotherapy (Tuesday to Friday), amifostine was given subcutaneously, as previously described (Koukourakis *et al*, 2000a). Patients were pretreated with 5 mg of oral Tropisetron (Navoban®, Novartis) 1–2 h before the subcutaneous injection of amifostine. Amifostine (500 mg flat dose) was diluted in 2.5 ml normal saline and was injected subcutaneously to the shoulder or abdominal area. The injection was repeated daily, 20 min before each radiotherapy fraction. Amifostine was injected with the patient being in a sitting position. Blood pressure monitoring (before and after injection) was performed once every week.

### Toxicities, treatment modification and supportive care

In case of grade 3/4 non-haematological toxicity, the dose of Caelyx® or Taxotere® was reduced by 50% or chemotherapy was interrupted, depending on severity of the adverse effect. Grade 2 neutropenia was to be immediately followed by GM–CSF support (400 μg s.c. every Saturday and Sunday). This supportive regimen, established in previously published studies of ours (Koukourakis *et al*, 1998b, 2001), effectively protected from neutropenia patients receiving concurrent chemo–radiotherapy using a high dose of carboplatin fractionated regimen or docetaxel/carboplatin chemotherapy. In the present study, the adoption of this GM–CSF regimen immediately following the documentation of a grade 2 neutropenia, would potentially allow the elimination of neutropenia as a dose limiting toxicity factor.

All types of severe non-haematological ‘in field’ chemo–radiotherapy related toxicities such as grade 3 mucosal (esophageal or oral/pharyngeal) toxicity, extensive ‘in-field’ moist skin desquamation with ulceration, severe pain or exertional dyspnea were to be followed by radiotherapy and chemotherapy interruption till recovery (regression of symptomatology to grade 1). Patients with mucositis were supported with non-steroid anti-inflammatory regimen (mainly nimesulide) and prophylactic oral anti-fungal therapy. Acute radiation pneumonitis was treated with antibiotics and steroids if that was deemed necessary. Any infection was treated with appropriate antibiotic therapy. Severe asthenia was followed by interruption of both radiotherapy and chemotherapy till recovery.

Caelyx®- and Taxotere®- related severe non-haematological toxicities (outside the radiation field) such as pneumonitis, severe erythrodysesthesia, severe allergic reactions or other less frequently expected complications were followed by chemotherapy interruption and treatment continuation with radiotherapy alone.

### Definition of the maximum tolerated dose

When in a cohort of patients three out of five of them expressed severe ‘in filed’ non-haematological toxicity that necessitated more than 1 week radiotherapy delay, the toxicity was considered as dose limiting (DLT). Similarly, haematological grade 3/4 toxicity in a cohort of three out of five cases (despite the support with GM–CSF) or other severe non-haematological drug related toxicity in at least three out of five patients also defined a DLT. The Taxotere®/Caelyx® dose level immediately before the level where the DLT was observed, was the maximum tolerated dose (MTD). In this dose level a total of 15 patients were to be recruited.

### Statistical analysis

The statistical analysis and graph presentation of survival curves was performed using the GraphPad Prism 2.01 (GraphPad®, San Diego, CA, USA, www.graphpad.com) package. Survival curves were plotted using the method of Kaplan and Meier. The Fisher's exact test or the unpaired two-tailed *t*-test was used to test differences between categorical variables as appropriate. A *P*-value <0.05 was used for significance.

## RESULTS

Twenty-five patients were recruited in the study. Five in dose level 1, five in dose level 2 and 15 in dose level 3, which was the target dose level . Table 2

**Table 2:**
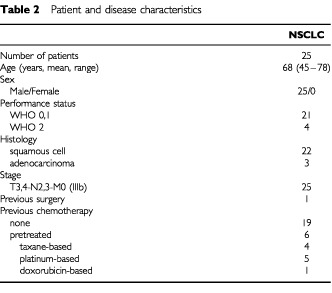
Patient and disease characteristics

shows the patients characteristics. Twenty-four had evaluable disease on CT-scan, while one had known residual (non-measurable) disease after incomplete surgery.

### Systemic toxicity of chemotherapy

In all three dose levels, the Taxotere®/Caelyx® chemotherapy combination showed an excellent tolerance. Grade 1 neutropenia was noted in five out of 15 patients treated in the 3rd dose level. Grade 2–4 neutropenia was not noted in the 25 treated patients, so that GM–CSF was not used. Grade 2 anaemia was noted in one out of 10 and two out of 15 patients in the 1st–2nd and the 3rd dose level. Patients were transfused with red cells till the Hb levels reached values >11 gr dl^−1^. No platelet toxicity was observed. The lymphocyte counts dropped from 1241±307 dl^−1^ down to 782±390 dl^−1^ during the 3rd week and to 749±245 during the 6th week of treatment.

Mild grade 1 palmar-plantar erythrodysesthesia related to Caelyx® was noted in two out of 15 patients treated in the 3rd dose level. Alopecia grade 1 was noted in 24 out of 25 and grade 2 in one out of 15 (3rd dose level) patients. No case of peripheral docetaxel related neuropathy, pulmonary, cardiac or renal toxicity was noted.

### ‘In-field’ toxicity

The main expected ‘in field’ acute non-haematological toxicity of the combination was esophageal mucositis. Grade 3 dysphagea appeared during the 3rd–4th week in two out of five, one out of five and two out of five patients in the three dose levels, respectively. Radiotherapy was interrupted and esophagitis regressed to grade 0/1 within 3–7 days in all patients. Two patients (one in the 1st and one in the 3rd dose level) showed fungal infection and were treated with oral fluconazole (100 mg three times-a-day for 7 days). None of the patients developed higher than grade 1 skin toxicity and there was no case with acute pneumonitis. Severe cough due to tracheitis, which is not rare during chest radiotherapy, was not observed in any of the patients.

As both systemic and ‘in-field’ toxicity were minor in the 3rd dose level, recruitment of patients in this latter level continued to reach a total of 15 patients. Grade 3 esophagitis appeared in six out of 15 (40%) patients, in four of which there was a superimposed fungal infection. A split of chemo–radiotherapy of 1 week was inserted for these patients. In four (26%) of these patients, esophagitis regressed to grade 1 following a 7-day radiotherapy split, while in the remaining two (13%) patients (both with fungal infection) an 8–14 day split was necessary. No other ‘in-field’ toxicity higher than grade 1 was noted. Although the dose limiting toxicity was not reached in the 3rd dose level, no further escalation of the dose of chemotherapy was attempted.

Within 18–24 months of follow-up, CT-scan findings of ‘in-field’ radiation lung fibrosis were detected in seven out of 25 (28%) patients but, there was not a case with symptomatic exertional dyspnea requiring oxygen support. No patient developed radiation-related soft tissue, neurological or cardiac late sequelae.

### Amifostine tolerance

Local subcutaneous injection of amifostine was associated with mild local pain, while extensive local erythema, necessitating the changing of multiple sited injections and local application of steroid cream, was experienced in one out of 25 patients (4%). Grade 2 nausea due to s.c. injection of amifostine was noted in two out of 25 (8%) patients. Hypotention was not observed in any of the 25 recruited patients. Of interest, the fever/rash symptomatology, which occurs in 8% of patients receiving s.c. amifostine (Koukourakis *et al*, 2000a), was also unobserved.

The intravenous administration of amifostine was well tolerated, with grade 2 nausea observed in three out of 25 (12%) of patients. Using the administration procedure described, symptomatic hypotention did not occur, but a significant drop of the systolic blood pressure to values <90 mm Hg, requiring 3–5 min infusion interruption (during one or more of the scheduled infusions) was necessary in 12 out of 25 patients (48%). Asthenia grade 2 (whether related to docetaxel or amifostine) was observed in three out of 25 (12%) patients. Therapy (chemotherapy and radiotherapy) was interrupted for 1 week in one patient. The dose of amifostine was reduced to 300 mg and two out of three patients completed the scheduled therapy. One patient presented fever (39°C) after the 4th i.v. injection and amifostine was discontinued.

### Comparative analysis of toxicity

The main in-field and systemic toxicities recorded in this study were comparatively analysed with the toxicities noted in three previous studies of ours in patients with locally advanced NSCLC treated with (1) conventionally fractionated chemo–radiotherapy with weekly Taxotere® 30 mg m^−2^ (Koukourakis *et al*, 1999c), (2) conventionally fractionated chemo–radiotherapy with Caelyx® once every 2 weeks 25 mg m^−2^ (Koukourakis *et al*, 1999b) or, (3) conventionally fractionated radiotherapy with and without subcutaneous administration of amifostine (Koukourakis *et al*, 2000a). The analysis is reported in Table 3

**Table 3:**
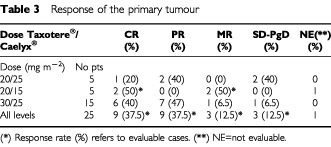
Response of the primary tumour

. Esophageal toxicity was impressively reduced. Esophagitis enforcing more than 1 week delay of radiotherapy was noted in 23–33% of patients treated with RT+Taxotere or RT+Caelyx without amifostine *vs* 12% in patients treated with RT alone *vs* 8–13% of patients treated with RT+Taxotere+Caelyx supported with amifostine.

Despite the administration of docetaxel, a drug associated with high incidence of profound asthenia, the asthenia noted in the present study was reduced compared to the docetaxel/RT study (12 *vs* 23% grade 2 asthenia). The typical fever/rash amifostine related symptomatology, noted in 10% of NSCLC patients treated with standard RT and s.c. administered amifostine was absent, although one patient presented fever without rash and amifostine administration was interrupted.

**Figure 1:**
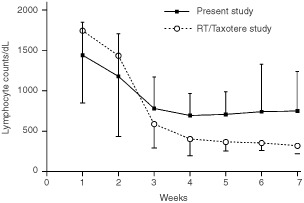
Comparative representation of the mean lymphocyte counts (per dl) and standard deviation in locally advanced non-small cell lung cancer patients treated in the present study (radiotherapy with Taxotere®/Caelyx® supported with amifostine) and in a previous phase II study of radiotherapy and Taxotere® (without amifostine).

No grade 2 neutropenia has been observed, compared to the 20% rate expected. Three and 6 weeks following the beginning of radiotherapy, the mean lymphocyte counts of patients in the present study were significantly higher compared to the ones recorded in the phase II study of Taxotere® and radiotherapy without amifostine (3rd week: 782±390 *vs* 589±297, *P*=0.009 and 6th week: 741±243 *vs* 354±93; *P*=0.0001). Figure 1

shows the lymphocyte counts obtained in the two studies.

### Response and outcome

Table 4

**Table 4:**
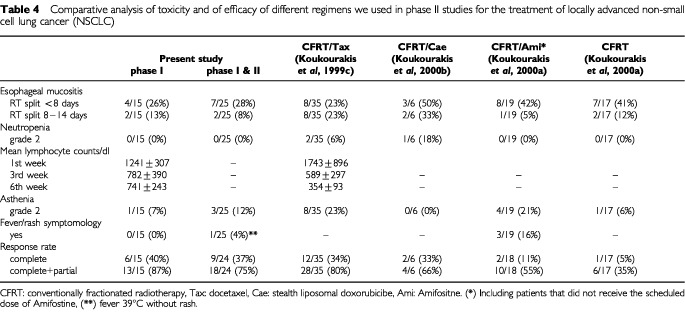
Comparative analysis of toxicity and of efficacy of different regimens we used in phase II studies for the treatment of locally advanced non-small cell lung cancer (NSCLC)

shows the responses observed, 2 months following RT, stratified by dose level. Complete response of the chest disease was observed in nine out of 24 (37.5%) and partial response in nine out of 24 (37.5%) evaluable cases, the total response rate being 75%. The overall response rate in the 3rd dose level was 87% (40% CR and 47% PR). Figure 2

**Figure 2:**
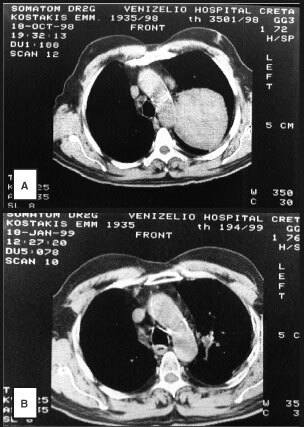
A CT-scan from a lung squamous carcinoma before (**A**) and after the end (**B**) of Taxotere®/Caelyx® chemo–radiotherapy.

shows the CT-scan of a large lung squamous cell carcinoma before therapy and its complete remission 2 months following the end of treatment.

The efficacy of the present schedule in terms of responses obtained compares favourably to previous regimens (Table 3). The criteria used for recruitment of patients in the present study were the same used in these historical studies and age, performance status and disease stage of patients recruited are similar in all studies reported in Table 3. Both the complete and the combined complete/partial response rates were significantly higher compared to the responses obtained in a previous study in locally advanced NSCLC treated with radiotherapy alone (CR 40 *vs* 4%; *P*=0.003 and CR/PR 87 *vs* 20%; *P*=0.0001) or with radiotherapy supported with subcutaneous amifostine administration (CR 40 *vs* 7%; *P*=0.01 and 87 *vs* 33%; *P*=0.001). The response rates were comparable to the ones obtained in a previous study on docetaxel chemo–radiotherapy and slightly higher than the ones obtained in a study with liposomal doxorubicine chemo–radiotherapy.

After 18–24 months of follow-up, seven (28%) patients are still alive with no evidence of local or distant disease. Eight patients died due to local progression, two of which had also distant metastases. Overall nine patients expressed distant metastases. One patient died due to the development of malignant pericardial effusion immediately after therapy, and one due to pulmonary embolism 1 month following treatment completion.

Figure 3

**Figure 3:**
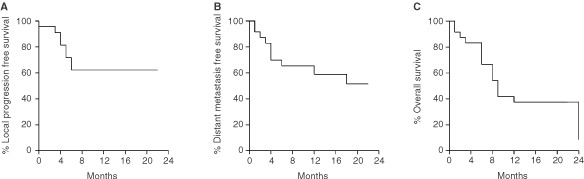
The Kaplan Meier local relapse free (**A**), distant relapse (**B**) free and overall survival (**C**) curves plotted for NSCLC patients recruited in the present phase II study.

shows the Kaplan–Meier survival curves of overall survival, local progression free survival and distant metastasis-free survival. The median survival was 9 months.

## DISCUSSION

The results of radiotherapy for inoperable non-small cell lung cancer (NSCLC) are frustrating. Concurrent or induction chemotherapy slightly improves the figures; median survival is marginally improved in randomized trials (Marino *et al*, 1995). Combination of novel drugs with radiotherapy is an interesting and promising field of clinical research. However, radiation toxicity becomes unacceptably high and may jeopardize the outcome of eventual phase III trials investigating such combinations. The establishment of well tolerated chemo–radiotherapy schemes is therefore essential before the initiation of randomized trials aiming to establish new standards for the treatment of locally advanced carcinomas.

In a previous randomized phase II study, we found that a simple subcutaneously administered schedule of amifostine significantly protected normal mucosa against radiation and prevented unnecessary delays that compromise the efficacy of radiotherapy (Koukourakis *et al*, 2000a). In the present study we investigated whether this broad-spectrum cytoprotective regimen can improve the tolerance of concurrent chemo–radiotherapy with high doses of two novel drugs (Taxotere® and Caelyx®) for NSCLC. Both drugs have important radiosensitizing properties. Taxanes induce synchronization of cancer cells to the radiosensitive G2/M phase of the cell cycle (Walf *et al*, 1996) and, furthermore, induce phosphorylation of the anti-apoptotic bcl-2 protein, which enhances radiation apoptosis (Haldar *et al*, 1996; Beham *et al*, 1998). Stealth liposomal doxorubicin is preferentially accumulated into the tumoural environment (Stewart and Harrington, 1997; Yuan *et al*, 1994; Harrington *et al*, 2001c) and the free-doxorubicine released has both cytotoxic and radiosensitizing properties (Bonner and Lawrence, 1990; Harrington *et al*, 2000). Moreover, the taxanes may further sensitize cancer cells to a DNA damaging agents, such as doxorubicine. In this way, multiple radio- and chemo- sensitization expected with such a regimen may result to an increased anti-tumour effect but, also to an increased toxicity.

In previous phase I/II studies we established the MTD of each one of these drugs for combination with radiotherapy (Koukourakis *et al*, 1998a, 1999a,b,c). Mucosa toxicity was high and unnecessary splits of radiotherapy were enforced, which may have reduced the efficacy of the regimens (Koukourakis *et al*, 1999c). In the present study, pre-treatment of patients with subcutaneous amifostine allowed the administration of full dose of both drugs in combination with conventionally fractionated radiotherapy. Comparative analysis of the mucositis observed in the present and in previous chemo–radiotherapy studies conducted with the same drugs but without amifostine, showed that the mucositis induced after high dose concurrent chemo–radiotherapy supported with subcutaneous amifostine is impressively reduced close to the one expected from standard radiotherapy alone. These results are encouraging as mucositis is the dose-limiting toxicity encountered in novel chemo–radiotherapy combinations and subcutaneous amifostine may therefore be a key for the succesful application of these novel regimens.

Similarly low was the incidence of acute radiation pneumonitis, which is quite common when radiotherapy is combined with taxanes (Reckzeh *et al*, 1996; Koukourakis *et al*, 1999c). Amifostine is well known to protect against radiation pneumonitis (Antonadou *et al*, 2001; Komaki *et al*, 2001). Skin toxicity was also low. These results, apart from confirming the important cytoprotective efficacy of amifostine, also eliminate worries on potential enhanced toxicity due to an eventual increased biodistribution of liposomal doxorubicine in irradiated normal tissues. Changes in vascular permeability and integrity induced by radiotherapy could result in increased accumulation of liposome within the irradiate lung, skin or muscles and end to destructive complications. Harrington *et al*, however, showed that radiotherapy does not change the normal tissue biodistribution and pharmacokinetics of liposomal doxorubicine, suggesting that combination of the drug with radiation should be safe (Harrington *et al*, 2001a,b). Indeed, the patterns of toxicity noted in the present study and in previous studies of ours (Koukourakis *et al*, 1999b, 2000b) combining Caelyx® with radiotherapy confirm that such regimens are safe for patients. Amifostine further improves the tolerability of such chemo–radiotherapy combinations.

Of interest, the incidence of asthenia, which is a main complication of docetaxel combination with radiotherapy was reduced. In previous studies we suggested that the intense immunosuppression related to docetaxel chemo–radiotherapy accounts for the asthenia of treated patients (Koukourakis *et al*, 1998a, 1999c). Protection of amifostine against immunological toxicity may therefore account for the reduced incidence of asthenia noted in the present study. Indeed, the lymphocytic toxicity noted in the present study was significantly less profound that the one noted in previous studies with docetaxel chemo–radiotherapy without amifostine.

Another provocative observation is that the fever/rash symptomatology related to amifostine, which is noted in 7–10% of patients undergoing protracted subcutaneous amifostine administration and consists a major cause of amifostine discontinuation (Koukourakis *et al*, 2000a; Shaw and Bleakley, 2000), was not observed in the present study. The fever/rash syndrome is not related to increased IgE levels or eosinophil counts, while a 2–3-fold increase of C-reactive protein is persistently noted in these patients, suggesting a cytokine/monokine release triggered by amifostine (Koukourakis *et al*, 2000a). Docetaxel chemotherapy or chemo–radiotherapy is associated with a dramatic reduction of B-cell, T-cell counts as well as natural killer counts (Koukourakis *et al*, 1998a, 1999a; Kotsakis *et al*, 2000). It may be, therefore, that the immunoreactive-cell count reduction induced by docetaxel accounts for the fever/rash symptomatology elimination. Nevertheless, the once-a-week high dose of methylprednisolone administered in the context of the pre-medication for the administration of docetaxel may also have contributed to cytokine release suppression. This finding provides a rational for testing whether steroids may improve the tolerance of subcutaneous amifostine. Once-a-week methylprednisolone 32 mg per o.s. is the suggested schedule of steroids. Our limited experience with patients exhibiting poor tolerance of subcutaneous amifostine (asthenia, vomiting) also shows that once-a-week injection of slow release cortisone indeed improves tolerability (unpublished data).

Despite the relatively high dose intensity of Taxotere® (120 mg m^−2^ every 4 weeks) and of Caelyx® (50 mg m^−2^ every 4 weeks), the combination of each one of these drugs separately with radiotherapy results in a low neutrophil toxicity. The grade 2 neutropenia expected from Caelyx® combination with radiotherapy at this dose intensity is in the range of 20%. In the present study, despite the double-chemotherapy scheme used together with radiotherapy, there was not a case of grade 2 neutropenia. This should be attributed to the protracted subcutaneous schedule of amifostine. Caelyx® has a long half-life of 92 h in the plasma, so that the i.v. dose of amifostine given before Taxotere®/Caelyx® infusion is unlikely to have protected the bone marrow from the cytotoxicity of Caelyx®. Indeed, in an experimental study in mice, i.v. injection of 100 mg kgr of amifostine for four consecutive days following the administration of liposomal doxorubicin, significantly reduced the rate of erythrodysesthesia without reducing the anti-neoplastic activity of the drug (Lyass *et al*, 2001). Erythrodysesthesia was negligible in the present study.

The response rate obtained was very high compared to the expected from simple conventionally fractionated radiotherapy, reaching 40% CR and 87% mixed CR and PR rates. However, similar response rates are also expected following docetaxel chemo–radiotherapy. Whether Taxotere®/Caelyx® chemo–radiotherapy is superior to Taxotere® chemo–radiotherapy is an issue that cannot be answered by the present study. Nevertheless, the excellent tolerance and the impressive CR rates obtained with this double chemosensitization regimen, despite the large tumour burden, provides the basis for subsequent relevant randomized trials.

It is concluded that the subcutaneous administration of amifostine during high dose Taxotere®/Caelyx® chemo–radiotherapy is a simple and effective way to render this aggressive regimen perfectly well tolerated by reducing the mucosal toxicity to levels expected from simple conventional radiotherapy. No haematological or other systemic toxicity is expected. Randomized trials with this promising double-radiosensitization regimen are warranted, to assess an eventual survival benefit in patients with inoperable locally advanced non-small cell lung carcinoma. The dramatic responses expected with such combinations also put forward an eventual re-assessment of patients for surgery, which should be also taken into account in the design of prospective trials.
